# Effects of changes to income tax and devolved benefits in Scotland on health inequalities: a modelling study

**DOI:** 10.1093/eurpub/ckaf009

**Published:** 2025-02-11

**Authors:** Elizabeth Richardson, David Walsh, Gerry McCartney, Andrew Pulford, Mark Robinson

**Affiliations:** ER and AP are Publishing in a Personal Capacity; School of Health and Wellbeing, University of Glasgow, Glasgow, United Kingdom; School of Health and Wellbeing, University of Glasgow, Glasgow, United Kingdom; ER and AP are Publishing in a Personal Capacity; School of Health and Wellbeing, University of Glasgow, Glasgow, United Kingdom; Institute for Social Science Research, The University of Queensland, Long Pocket Precinct, Indooroopilly, QLD, Australia

## Abstract

There is a well-understood relationship between inequalities in income and health. We assessed how changes to income tax and social security—options recently devolved to the Scottish Government—could affect income and life expectancy inequalities. We used the microsimulation model UKMOD to estimate policies’ effects on household income distribution by socioeconomic deprivation, compared to baseline (Scottish income tax schedule for 2022/23). We then used the ‘Triple I’ (Informing Interventions to reduce health Inequalities) scenario modelling approach to estimate mortality effects for the income changes and calculated inequalities in life expectancy at birth. Scenario health impacts were determined largely by how much money they gave or took from households in the most deprived areas. Policies that increased incomes for households in deprived areas tended to reduce inequalities in life expectancy. Although we found this also applied to tax-cutting policies that increased income inequality, our estimates did not account for the public spending cuts that these costly policies would necessitate and their likely widening effect on health inequalities. Combining the best-performing (i.e. greatest positive impact) revenue-generating and revenue-spending policies we modelled—tax increases targeted at high earners and a doubling the value of social security benefits—would generate net revenue while reducing income inequality by approximately 10% and inequalities in life expectancy by 8% to 9%, but sizeable inequalities would remain. A multifaceted approach based on combinations of policies—including, but not limited to, bolder income tax measures—is required to achieve meaningful reductions in inequalities.

## Introduction

Reducing health inequalities has been a stated aim of many governments, including several in the UK. ‘Fundamental causes’ [1, 2] of health inequalities are socioeconomic: thus, the use of economic policy to narrow inequalities is of profound importance.

In Scotland, the desire to narrow economic and health inequalities has been emphasized by the devolved government [3]. However, with the majority of the most relevant economic powers being reserved to the UK (Westminster) Parliament, the Scottish Government’s ability to narrow inequalities in this area has been limited. To a limited degree, this changed with the Scotland Act 2016 which devolved new powers over income tax rates and bands, and a small number of social security benefits, to the Scottish Parliament [4]. Income tax and social security benefits are two key governmental levers available to redistribute income more equitably and, in the case of income tax, to generate revenue for public services. Understanding the potential impact of these levers on health inequalities is of increasing importance in light of the current cost of living crisis and its implications for public health [5].

We use scenario modelling [6] to assess the impact of implemented and potential changes to income tax rates and bands, and devolved social security benefits, on household incomes, income inequality, government revenue, and, ultimately, health inequalities. We use Scotland as a case study, but the research is clearly relevant to other administrations seeking to narrow societal inequalities.

## Methods

### The policy scenarios

We modelled 13 implemented or proposed variations in income tax rates and bands (Supplementary Appendix S1). The baseline scenario, against which the effects of the other scenarios were compared, consisted of the rates and bands set by the Scottish Government for 2022/23. We included all alternative bands and rates proposed in the manifestos of opposition parties in the 2016 and 2021 Scottish Parliament elections (Supplementary Appendix S2). The other scenarios were recent or current regimes implemented by Scottish or UK Governments. After performing the tax-benefit modelling, the scenarios were named sequentially in order of the revenue they would generate: from low (those with a net cost) to high (net revenue). Powers over tax-free personal allowance (PA) remain reserved to Westminster, and all but one proposal used the default PA for that year: £11 500 in 2017/18, £12 500 in 2019/20, or £12 570 in 2022/23. To ensure comparability between scenarios proposed in different years, we set the PA as £12 570 for scenarios using the default PA (Supplementary Appendix S1).

We also modelled increases to the rates of the main social security benefits that had been devolved to or created by the Scottish Government at the time of the analysis (Supplementary Appendix S3). We modelled increases of 10%, 25%, 50%, 75%, and 100% in their rates, with income tax maintained as per the baseline scenario.

### Policy effects on household and government incomes

We estimated the impact of each policy on household incomes in Scotland in 2022/23, grouped by Scottish Index of Multiple Deprivation (SIMD) quintile (fifth). We used the tax-benefit microsimulation model UKMOD (version A3·23+) [7], to estimate incomes (before housing costs) for Scottish households in the Family Resources Survey (see Supplementary Appendix S4 for details).

We estimated the level of income inequality that would directly result from each policy by calculating the Gini coefficient. Preliminary estimates of direct policy costs to the government were calculated from the effects on household tax bills and benefits receipts, grossed up to the national level using the relevant weights. The net relative cost of each policy was estimated by subtracting its cost from that of the baseline scenario.

### Policy effects on inequalities in life expectancy

We then estimated how the income change experienced within each SIMD quintile under each scenario was likely to affect life expectancy, using the Informing Interventions to reduce health Inequalities (‘Triple I’) approach [6]. This is an existing scenario modelling approach that has been applied previously in Scotland [6, 8].

There is evidence that income change is related to mortality risk [9, 10] but no generalizable effect size is available [11]. We therefore estimated the relationship for Scotland, using cross-sectional data (Supplementary Appendix S5). The function choice was informed by evidence that marginal income differences matter more for the physical and mental health of those on lower incomes [12, 13]. As the mortality effect size calculation is a strong assumption, we conducted a sensitivity analysis by reducing the effect by 50%.

Using the estimated deaths distribution from the Triple I model for each scenario, we calculated life expectancy by deprivation quintile (SIMD). We calculated inequality in life expectancy, using the slope index of inequality (SII) and relative index of inequality (RII). The SII and RII are linear regression-based indices that account for absolute and relative health differences, respectively, across the whole socioeconomic gradient [14].

### Role of the funding source

The study design, data collection, data analysis, data interpretation, and writing of the report were not influenced by funders.

## Results

**Figure 1.**
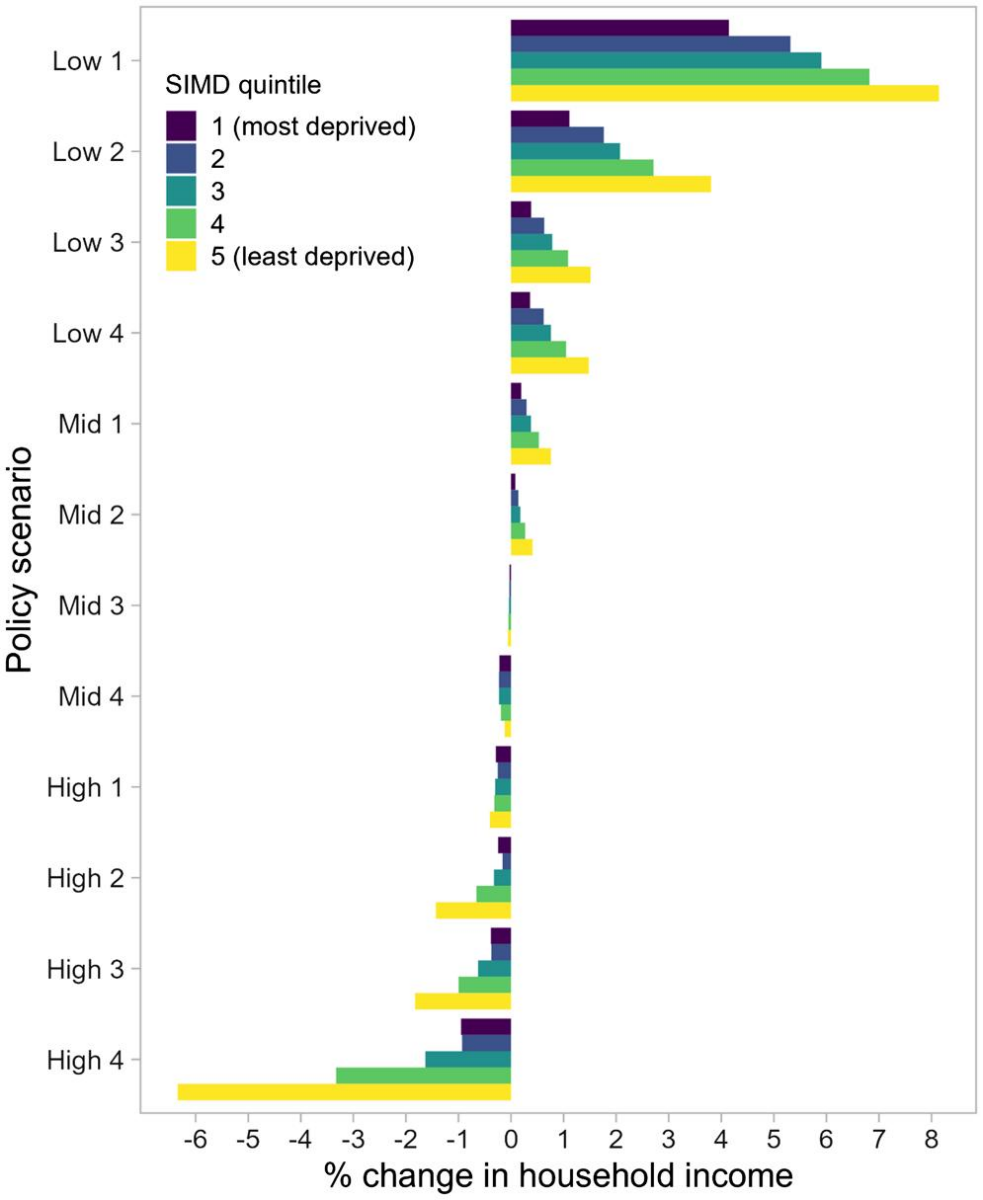
Effects on household incomes of changes to income tax, compared to the baseline ‘no-change’ scenario, by SIMD quintile, 2022/23. Scenarios are ranked in order of the revenue they would generate: from low (those with a net cost) to high (those with a net revenue).

**Figure 2.**
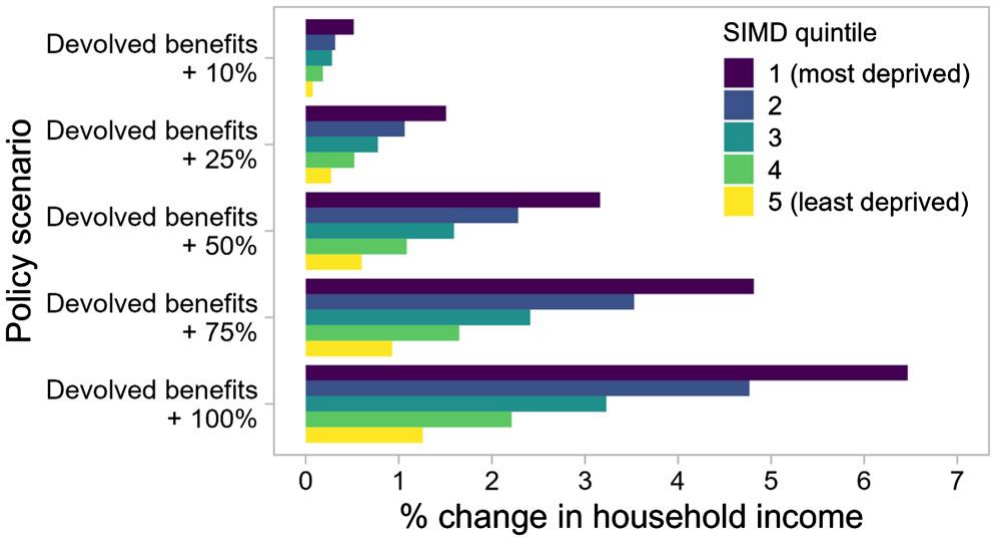
Effects on household incomes of changes to devolved benefits, compared to the baseline ‘no-change’ scenario, by SIMD quintile, 2022/23.

The estimated effects of the fiscal changes on household incomes by SIMD quintiles, compared to the baseline scenario, are shown in Figs 1 and 2.

Most income tax policies we modelled caused extremely modest changes in household income: 9 out of 12 led to changes of less than 2%. The four most costly income tax schedules—‘Low 1’ to ‘Low 4’—cut taxes for higher earners and would reduce average tax bills across all deprivation quintiles (resulting in increased household income) (Fig. 1). Under these policies, household incomes would increase least for those in the most deprived areas and most for those in the least deprived areas.

At the other end of the spectrum, the four scenarios generating the most revenue—‘High 1’ to ‘High 4’—would increase tax bills across the population in a more progressive way, by increasing the rates applied to higher earners. As a result, incomes would fall most for those in the least deprived areas and least for those in more deprived areas (Fig. 1). The ‘High 4’ scenario—which increased income tax rates to 90% for the highest earners—would result in the largest narrowing of income differences across SIMD quintiles, with seven-fold higher income reduction in the least deprived areas (6.3%) compared with in the most deprived areas (0.9%). All deprivation quintiles would experience household income reductions because each will contain earners from different tax brackets.

Increases to devolved benefits would result in increased household income across all deprivation quintiles (Fig. 2). Five of the 12 benefits we modelled are means-tested (i.e. paid to those below an income threshold); hence, there is a socioeconomic gradient in claims. Consequently, increases would have the biggest effects on incomes in the most deprived areas: proportional increases here would be more than five times those for the least deprived areas.

**Figure 3.**
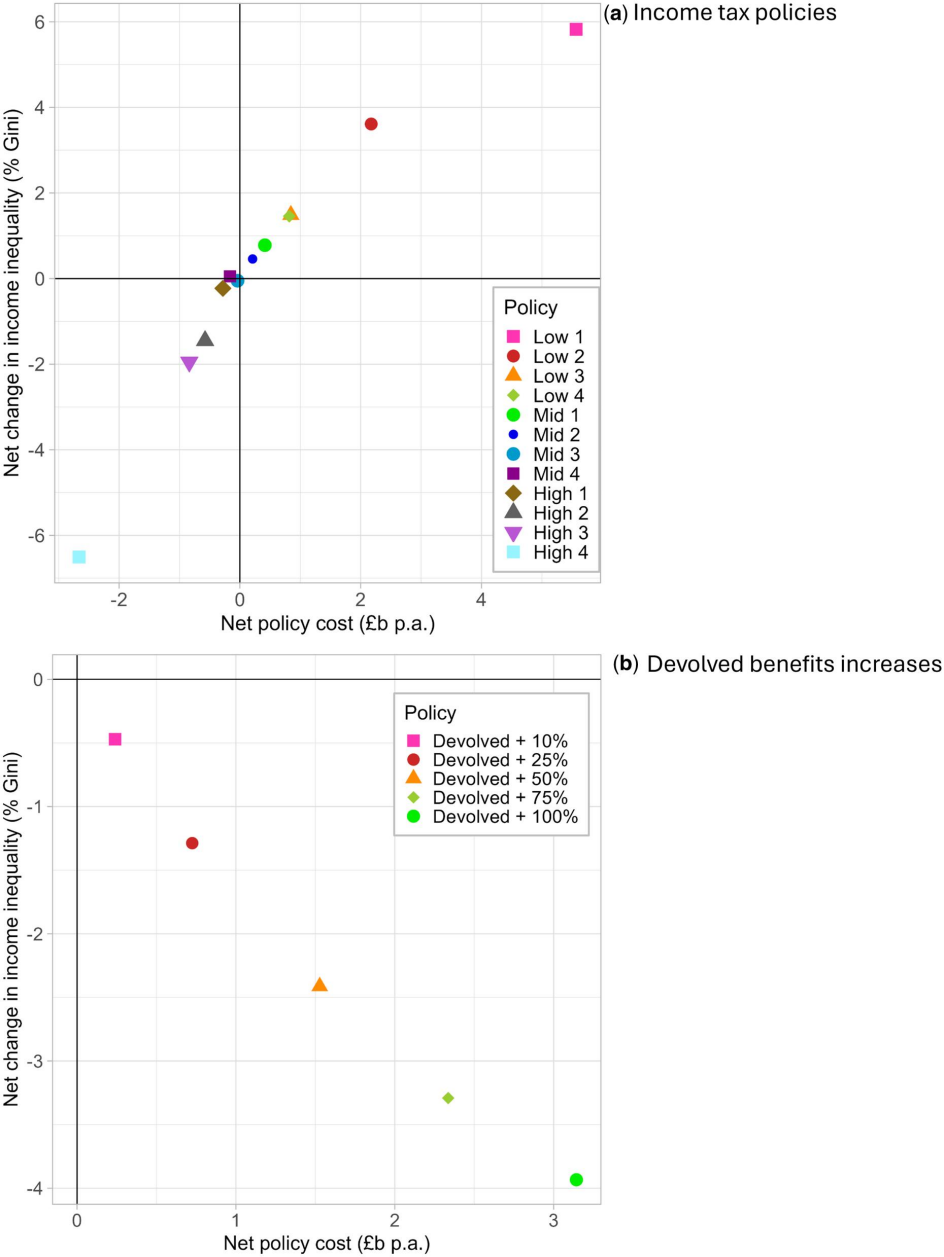
Implications of the policy scenarios for net cost to government and income inequality, compared with the baseline ‘no change’ scenario (Scotland in 2022/23) for (a) income tax policies and (b) devolved benefits increases. Negative values indicate reductions (i.e. lower cost or lower income inequality).

The relative costs of each policy, and their predicted effects on income inequality in the Scottish population, varied in line with the household-level effects (Fig. 3, Supplementary Appendix S6). Policies that increased household incomes (whether through reduced tax bills or increased benefits) would cost the government more than the baseline scenario, and those that decreased household incomes would represent a net saving, in relative terms. Policies observed in Fig. 1 to affect those in the least deprived areas more favourably than those in the most deprived areas would increase income inequality, while the policies with a more progressive design, and the benefits increases, would reduce income inequality. Scenario ‘Low 1’—the scenario with the largest tax cuts—would cost an estimated £5.6 billion more than baseline and increase income inequality by 5.8%. Scenario ‘High 4’ would represent the largest net saving (£2.6 billion) and result in an estimated 6.5% reduction in baseline income inequality. The doubling of devolved benefits would reduce income inequality by 3.9% (Fig. 3).

Life expectancy at birth in the baseline scenario (based on 2021 deaths and population) was estimated as 80.6 years for females and 76.5 years for males (Supplementary Appendix S7). The RII of 0.20 for male life expectancy at birth means that the range between life expectancy in the most and least deprived areas is approximately 0.20 times the population average life expectancy (76.5 years), or 15.3 years.

**Figure 4.**
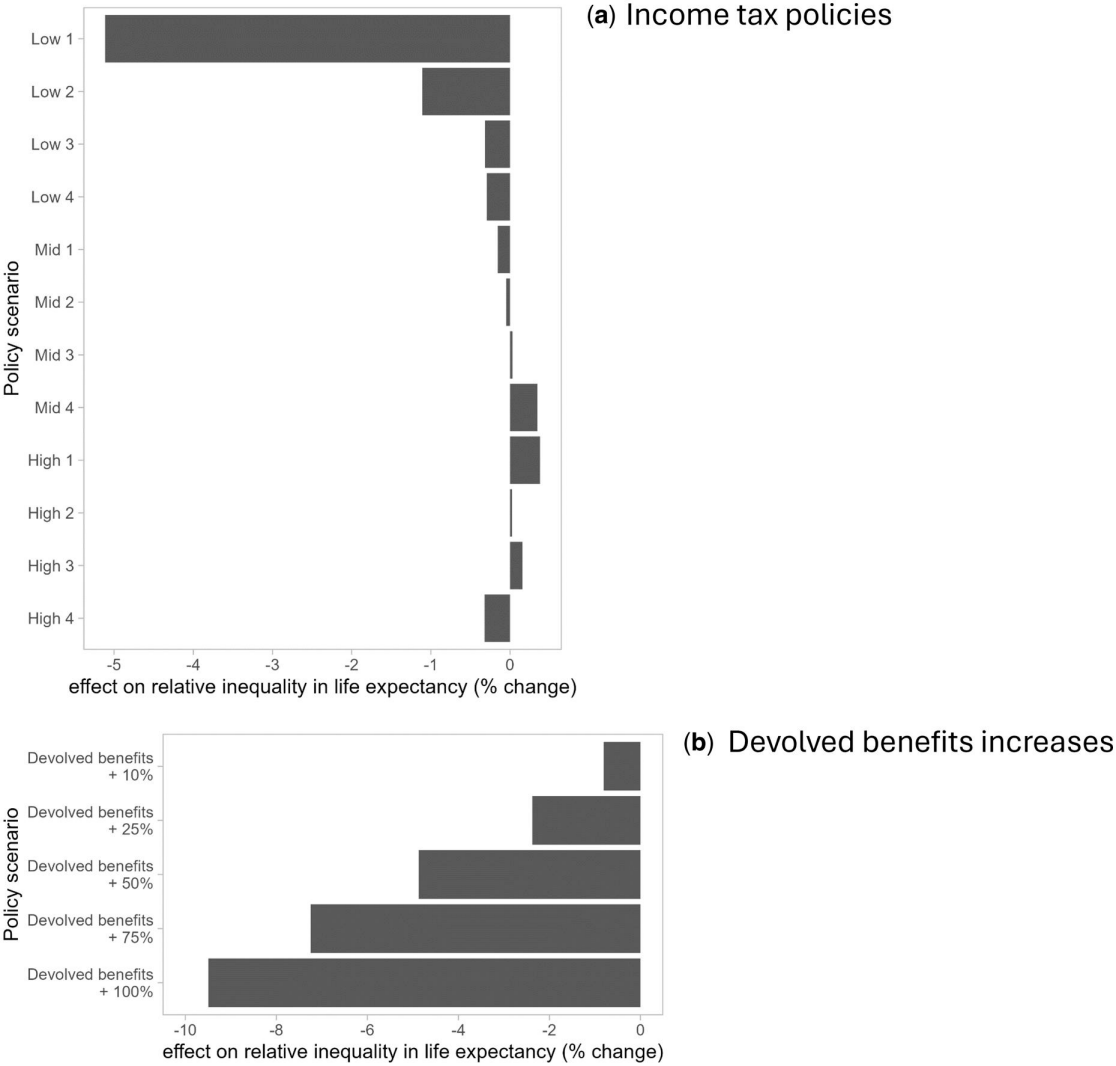
Policy effects on relative inequalities in female life expectancy, 2022/23. Results shown for (a) income tax policies and (b) devolved benefits increases. Relative inequality measured by relative index of inequality (RII). Change measured relative to the baseline ‘no change’ scenario.

Each scenario’s impacts on life expectancy (Supplementary Appendix S8) and life expectancy inequalities (Fig. 4, Supplementary Appendix S8) were driven by its effect on incomes in more deprived areas. This was because our model assumed the same change in income would have a bigger impact on mortality for lower income than higher income households (Supplementary Appendix S4). Scenarios that increased incomes in more deprived areas were estimated to reduce inequalities in life expectancy, and those that decreased incomes in these areas were estimated to increase inequalities, regardless of their effects in less deprived areas (Fig. 4 for relative inequalities, Supplementary Appendix S8 for absolute inequalities). Thus, by introducing substantial tax cuts, the most expensive scenario—‘Low 1’—would be estimated to reduce relative inequality in life expectancy the most (5.1% female, 4.7% male), largely because it increased incomes in the most deprived areas more than other scenarios did (4.1%), leading to an estimated 30 week gain in life expectancy. Although this scenario resulted in higher income increases in less deprived areas (e.g. 8.1% in the least deprived areas), these were predicted have a smaller impact on life expectancy (e.g. 7 week gain in the least deprived areas), and hence, inequalities would be predicted to narrow.

Of the four more progressive and revenue-generating policies (‘High 1’ to ‘High 4’), only ‘High 4’ was estimated to reduce inequalities in life expectancy (relative: 0.3% female, 0.1% male; absolute: 0.5% female and 0.3% male) (Fig. 4, Supplementary Appendix S8). The discrepancy in income effects under this scenario (Fig. 1) would be large enough to result in smaller life expectancy reductions in the most deprived areas (7 weeks) than in the least deprived areas (8 weeks). For all of these results which model the impact of tax changes alone, and particularly the ‘Low’ scenarios which incur a high cost to government, it must be noted that we do not include the consequences this is likely to have for public spending, including on social security benefits. If the ‘Low’ scenarios are accompanied by cuts to social security and public services (which in turn would be likely to reduce wages and service availability), the impacts on mortality inequalities would be likely to reverse in direction.

Increases to devolved benefits would reduce inequalities in life expectancy (Fig. 4, Supplementary Appendix S8). The extent of the reduction would be dependent on the size of the increase (and thus the level of investment): a 10% increase (costing £0.2 billion p.a.) would be estimated to result in a 0.8% reduction for females (0.7% for males), while a doubling of the benefits (costing £3.1 billion p.a.) could reduce inequalities by 9.5% for females, and 8.4% for males.

Comparisons of net cost of the different policies and their likely impact on life expectancy inequalities are shown in Supplementary Appendix S9. These confirm that policies with a high net cost could potentially reduce life expectancy inequalities (but only if, as stated above, the costs to government did not limit public spend elsewhere), whereas the highest revenue policy (‘High 4’) could reduce inequalities while also saving the government money.

We modelled single policies so that their effects on household incomes could be demonstrated, but in reality, policies would be implemented concurrently. Increased revenues from taxation could be used to increase public spend, or the shortfall arising from decreased tax revenue, or increased benefit spend, could reduce public spend elsewhere. We therefore modelled the effects of combining the single income-generating taxation policy that narrowed inequalities (‘High 4’) with increases to devolved benefits.

The ‘High 4’ income tax schedule would fully cover the cost of doubling devolved benefits and would generate an additional £40 million in revenue, compared to baseline (Supplementary Appendix S10). Compared with baseline, under this combined scenario, household incomes in the more deprived SIMD quintiles would increase and those in the less deprived quintiles would decrease (Supplementary Appendix S10). Female life expectancy would be estimated to increase by 36 weeks in the most deprived areas (40 weeks for males; Supplementary Appendix S11) and decrease by 7 weeks in the least deprived areas (7 weeks for males). As a result, relative inequality in life expectancy would decrease by 9.4% for females and 8.1% for males (Supplementary Appendix S11).

We found that the same relative changes to household incomes would have a much larger effect on life expectancy in more deprived than in less deprived areas, with effects being between seven and nine times larger in the most deprived than the least deprived areas (Supplementary Appendix S5, Table A2).

In our analysis of the sensitivity of the results to the strength of the relationship between income change and mortality rates (Supplementary Appendix S12), effect sizes were reduced by approximately 50%, although their directionality and relative positions were unaffected.

## Discussion

We modelled the effects of changes to income tax and devolved social security benefits, on income, life expectancy, and inequalities in life expectancy. The estimated effects of most income tax scenarios were very modest. The direct income changes resulting from the most costly scenario—‘Low 1’—were estimated to increase income inequalities the most (around 6%) but also to reduce relative inequalities in life expectancy the most (around 5%). As a result of the tax cuts in this scenario, more deprived areas would experience smaller increases in income (in both relative and absolute terms) than less deprived areas, but these smaller income increases would result in larger predicted health gains. This is because income changes matter more for the health of those on lower incomes than those on higher incomes [12, 13]. However, in the context of the current devolution settlement for Scotland, these tax cuts would have to be accompanied by substantial cuts in public spending, which would impact in the opposite direction. This is important, as cuts to public services and social security benefits implemented since 2010 have been clearly evidenced to have large detrimental impacts on overall life expectancy trends and inequalities in life expectancy [15].

The highest revenue-generating scenario—‘High 4’—was by far the most progressive, but its direct impact on household incomes was estimated to result in only a modest 0.3% reduction in life expectancy inequality. Revenue generated under this scenario would enable greater public spending, with the potential to further narrow health inequalities.

Increasing the value of devolved social security benefits had greater effects, but only with large (and costly) increases. Combining the best-performing (i.e. greatest positive impact) revenue-spending and revenue-generating policies we modelled—a doubling of the value of social security benefits alongside implementing scenario ‘High 4’—would generate net revenue while reducing income inequality by approximately 10% and inequalities in life expectancy by 8%–9%.

These relatively modest effect sizes—particularly in relation to income inequality—are an important finding. Inequalities in mortality closely track inequalities in income: both were at an historic low in the late 1970s [16], when a quite different set of political and economic structures were in place. Income inequality was 30% higher in our baseline scenario (2022/23) than in the late 1970s; thus, for governments to reduce inequalities back to former levels, they would need to introduce not only much bolder and more progressive income tax policies but do so alongside a broad set of additional policies, such as changes in other taxation (e.g. corporation, wealth, and assets), changes to employment law, poverty reduction measures, a more protective social security system, and policy to reduce inequalities in pay and ownership of capital [17]. In Scotland’s case, this again raises the issue of whether—despite impressive policy statements—the Scottish Government currently has the necessary powers to bring about meaningful reductions in inequalities across society [18], although there are options particularly concerning current local taxation arrangements (council tax) [19].

The greater impact on inequalities of changes to social security payments compared to income tax, as well as the greater benefits of the introduction of ‘novel’ policies such as a Citizen’s Basic Income, has been demonstrated [6]. More generally, our study adds to other recent research that utilizes robust modelling techniques to examine the impact of particular policies where evidence is lacking [20]. Other modelling which uses panel data to estimate the relationship between income and employment changes and (mental) health outcomes suggests that employment changes have greater impacts than income changes [13].

The ability to understand the likely effects of policy changes in the absence of other evidence is one of the key strengths of this work. We used a representative sample of Scottish households, and thus, the results are applicable to the country as whole. Although particularly relevant to Scottish policymakers, the findings are also pertinent to other governments and add to the evidence for policies that can narrow health inequalities [21, 22]. We modelled policies in isolation, to quantify their individual contributions, as well as looking at more realistic policy combinations. The modelling tool used has been carefully developed and peer reviewed [6, 8].

The most obvious limitation of the work relates to the various assumptions that apply to all such modelling exercises. The assumption of the strength of the relationship between income and health has been discussed elsewhere [6], and sensitivity analyses were conducted to quantify the impact of this assumption on the results. This assumption arises from the lack of good data on the health impacts of income policy changes rather than from changes in employment or income flux within populations. The calculation of net government cost excluded health-related costs such as lost productivity and associated tax revenue, as well as healthcare costs: however, in the absence of additional data that would be required to undertake a more comprehensive health economics analysis, these estimates were intended to provide a comparative guide for policy makers about policy options.

We also examined the political parties’ income tax proposals in isolation from their other policy proposals. For example, for governments that raise tax revenues, any impacts on life expectancy inequalities will also be affected by how that additional revenue is spent (and vice versa). With the exception of social security payments, we could not model the impacts of additional public spending which may offset the loss of income due to higher income taxes. We only modelled the direct impact of changes in income rather than any indirect pathways. This may underestimate the impact of policies that narrow income inequality [23].

The policies may also have unintended consequences that we have been unable to account for in our modelling. For example, it could be argued that benefits increases might disincentivize work and prevent recipients getting the health benefits of employment. However, a review of evidence for the effects of unconditional cash transfers found little evidence that these resulted in reduced employment [24]. Similarly, it has been argued that increased income taxes, particularly on high earners, can lead to out-migration, reduced working hours, or tax evasion/avoidance, which might reduce the actual revenue raised. In Scotland, much of the high-earning workforce is in the public sector and is therefore less mobile than in other sectors. However, even in states dominated by the private sector, there is little evidence for out-migration of high earners [25, 26].

Some may argue that some of the income tax proposals are unrealistic. However, there are very substantial differences in income tax rates over time and between countries, reflecting different approaches to government revenue raising and different ideologies. It is worth noting that the UK had much higher top rates of income tax prior to the 1980s, sitting at 83% for much of the 1970s [27].

Our analysis also predates much of the increase in price inflation and the more modest increase in wages that occurred in 2022 and 2023. This is likely to have had its own impacts on health through changes in real incomes [8] but has also moved more wage-earners above each of the tax thresholds, increasing the tax revenues through ‘fiscal drag’. In the Scottish Government budgets since we conducted our analysis, the income tax rate paid by the highest earners has been increased and the threshold above which this is payable has been lowered. As of 2024/25, a top rate of 48% is payable for incomes over £125 140 [28].

Health inequality monitoring tends to use area deprivation measures to rank populations because the underlying data are readily available and easier to update. However, most income- and employment-deprived individuals do not live in the most deprived areas, and so changes to the income distribution have more muted impacts on deprivation-ranked populations because of this ecological mismatch [29]. Tax and benefit policies are also limited in the extent to which they address the economic relationships between social groups, such as rent and profit flows. A more comprehensive policy package would address these issues [30].

## Conclusion

The study provides further evidence of the likely effects of socioeconomic policy changes on income and health-related outcomes. The principal implication—that to achieve meaningful reductions in inequalities requires a multifaceted policy approach rather than single (e.g. income tax) policy changes—is an important one, not just for government in Scotland, but for all administrations seeking a narrowing of unfair and unjust divisions in society.

## Supplementary Material

ckaf009_Supplementary_Data

## Data Availability

The data underlying this article are available in the article and in its online supplementary material. Key points There is a clear relationship between inequalities in income and health, and inequalities in income can be modified via different taxation and social security policies. Powers allocated to the devolved Scottish Government in 2016 mean that different income tax bands and rates are now in operation in different parts of the UK: their likely impacts on health inequalities have not previously been explored. We compare the impact of 13 income tax policies, and 5 potential changes to social security, on income and health inequalities in Scotland. We demonstrate that impacts of the modelled policy changes are very modest. Thus, for governments to achieve meaningful reductions in health inequalities in the UK, they would need to introduce not only much bolder, more progressive income tax policies, but do so alongside a broad set of additional policies.

## Notes

**EUPHA membership**: David Walsh, Membership no. 12672
