# Supplementary material for: Effects of changes to income tax and devolved benefits in Scotland on health inequalities: a modelling study
Source: Eur J Public Health. 2025 Feb 11;35(2):242–8. doi: 10.1093/eurpub/ckaf009 (PMC11967894; doi:10.1093/eurpub/ckaf009)
Supplement: ckaf009_Supplementary_Data [file ckaf009_supplementary_data.docx]

**Effects of changes to income tax and devolved benefits in Scotland on health inequalities: a modelling study**

**Corresponding author:**

Dr David Walsh, [david.walsh.2@glasgow.ac.uk](mailto:david.walsh.2@glasgow.ac.uk)

**SUPPLEMENTARY MATERIAL**

**Contents**

[Appendix 1: Income tax scenarios 2](#_Toc184717641)

[Appendix 2: Sources of income tax schedule information (implemented and proposed) 3](#_Toc184717642)

[Appendix 3. Rates of social security payments devolved to the Scottish Government (2022/23). 4](#_Toc184717643)

[Appendix 4. Household income modelling 5](#_Toc184717644)

[Appendix 5. Effect of household income change on mortality rates 6](#_Toc184717645)

[Appendix 6. Estimated direct net fiscal costs of the scenarios, relative to baseline. 8](#_Toc184717646)

[Appendix 7. Life expectancy at birth, and inequalities in life expectancy at birth, in the baseline scenario (2022/23). 9](#_Toc184717647)

[Appendix 8. Differences in life expectancy and inequalities in life expectancy from the baseline scenario, by sex. 10](#_Toc184717648)

[Appendix 9. Implications of the policy scenarios for net cost to government and female life expectancy inequality. 11](#_Toc184717649)

[Appendix 10. Difference from baseline in scenario cost and income effects, for “High 4” and devolved benefits increases separately and in combination. 13](#_Toc184717650)

[Appendix 11. Difference from baseline in scenario life expectancy and life expectancy inequality effects, for “High 4” and devolved benefits increases separately and in combination. 14](#_Toc184717651)

[Appendix 12. Differences in life expectancy and inequalities in life expectancy from the baseline scenario, by sex, if the income-mortality relationship was weakened by 50%. 15](#_Toc184717652)

Appendix 1: Income tax scenarios

| **Scenario** | **Detail** | **Personal Allowance** | **Band 1** | **Band 2** | **Band 3** | **Band 4** | **Band 5** |
| --- | --- | --- | --- | --- | --- | --- | --- |
| Baseline | Income | £0 - £12,570 | £12,571 - £14,732 | £14,733 - £25,688 | £25,689 - £43,662 | £43,663 - £150,000 | >£150,000 |
|  | Tax rate | 0% | 19% | 20% | 21% | 41% | 46% |
| Low 1 | Income | £0 - £20,000 | £20,001 - £70,000 | >£70,000 |  |  |  |
|  | Tax rate | 0% | 20% | 40% |  |  |  |
| Low 2 | Income | £0 - £12,570 | £12,571 - £50,270 | >£50,270 |  |  |  |
|  | Tax rate | 0% | 19% | 40% |  |  |  |
| Low 3 | Income | £0 - £12,570 | £12,571 - £50,000 | £50,001 - £150,000 | >£150,000 |  |  |
|  | Tax rate | 0% | 20% | 40% | 45% |  |  |
| Low 4 | Income | £0 - £12,570 | £12,571 - £45,300 | £45,301- £55,000 | £55,001 - £150,000 | >£150,000 |  |
|  | Tax rate | 0% | 20% | 30% | 40% | 45% |  |
| Mid 1 | Income | £0 - £12,570 | £12,571 - £45,000 | £45,001 - £150,000 | >£150,000 |  |  |
|  | Tax rate | 0% | 20% | 40% | 45% |  |  |
| Mid 2 | Income | £0 - £12,570 | £12,571 - £43,000 | £43,001 - £150,000 | >£150,000 |  |  |
|  | Tax rate | 0% | 20% | 40% | 45% |  |  |
| Mid 3 | Income | £0 - £12,570 | £12,571 - £14,549 | £14,550 - £24,944 | £24,945 - £43,430 | £43,431 - £150,000 | >£150,000 |
|  | Tax rate | 0% | 19% | 20% | 21% | 41% | 46% |
| Mid 4 | Income | £0 - £12,570 | £12,571 - £45,000 | £45,001 - £150,000 | >£150,000 |  |  |
|  | Tax rate | 0% | 21% | 41% | 46% |  |  |
| High 1 | Income | £0 - £12,570 | £12,571 - £45,000 | £45,001 - £150,000 | >£150,000 |  |  |
|  | Tax rate | 0% | 21% | 41% | 50% |  |  |
| High 2 | Income | £0 - £12,570 | £12,571 - £43,500 | £43,501 - £61,500 | £61,501 - £150,000 | >£150,000 |  |
|  | Tax rate | 0% | 20% | 40% | 45% | 60% |  |
| High 3 | Income | £0 - £12,570 | £12,571 - £19,000 | £19,001 - £43,000 | £43,001 - £150,000 | >£150,000 |  |
|  | Tax rate | 0% | 18% | 22% | 43% | 60% |  |
| High 4 | Income | £0 - £12,570 | £12,571 - £43,000 | £43,001 - £90,000 | >£90,000 |  |  |
|  | Tax rate | 0% | 20% | 40% | 90% |  |  |

Appendix 2: Sources of income tax schedule information (implemented and proposed)

Twelve income tax scenarios were modelled and compared against the baseline (the bands and rates set by the Scottish Government for 2022/23). Two were the Scottish Government’s income tax regimes in 2017/18 and 2019/20; six scenarios were the proposals from opposition parties in the 2016 Scottish Parliamentary elections; one was the bands and rates applied in the rest of the UK by the UK Government in 2019/20; one was the UK Government’s proposed rates and bands for 2023/24 (which were subsequently scrapped); and two were proposed by parties in the 2021 Scottish Parliamentary elections. Only two parties’ proposals were included from 2021; other parties (including the largest) did not suggest changes because of a stated desire to focus on recovery from the COVID-19 pandemic. Details of the bands and rates (Table 1) were extracted from the following government sources or party manifestos:

Scottish Government (2017) Income tax rates set. <https://news.gov.scot/news/income-tax-rates-set> Accessed 16.1.2023.

Scottish Government (2018) Scottish income tax: 2019-2020 <https://www.gov.scot/publications/scottish-income-tax-2019-2020/> Accessed 16.1.2023.

UK Government (2016) Tax and tax credit rates and thresholds for 2017-18. <https://www.gov.uk/government/publications/tax-and-tax-credit-rates-and-thresholds-for-2017-18/tax-and-tax-credit-rates-and-thresholds-for-2017-18> Accessed 16.1.2023.

UK Government (2021) Income tax rates and allowances for current and past years. <https://www.gov.uk/government/publications/rates-and-allowances-income-tax/income-tax-rates-and-allowances-current-and-past> Accessed 16.1.2023.

Scottish Conservative & Unionist Party (2016) A strong opposition - a stronger Scotland. Scottish Conservative & Unionist Party Manifesto 2016. <http://www.spokes.org.uk/wp-content/uploads/2016/03/Scottish-Conservative-Manifesto_2016-DIGITAL-SINGLE-PAGES.pdf> Accessed 16.1.2023.

Scottish Labour Party (2016) Invest in Scotland’s Future – Both Votes Labour. Manifesto 2016. <http://www.spokes.org.uk/wp-content/uploads/2016/03/Scottish-Labour-Manifesto-2016.pdf> Accessed 16.1.2023.

Scottish Greens (2016) Fair funding for public services: Scottish Greens Taxation Proposals. <https://greens.scot/sites/default/files/Policy/Fair%20Funding%20For%20Public%20Services%202016.pdf> Accessed 16.1.2023.

Scottish Liberal Democrats (2016) Be the best again: Scottish Parliament 2016 Manifesto. <https://d3n8a8pro7vhmx.cloudfront.net/no2nuisancecalls/pages/1979/attachments/original/1460714587/Manifesto_-_Be_The_Best_Again-_Scottish_Liberal_Democrats_2016.pdf?1460714587> Accessed 16.1.2023.

RISE (2016) Another Scotland is possible. RISE Manifesto 2016. <http://static1.squarespace.com/static/56ab5f8e3b0be305046eca97/t/571636edf85082a0a4ed1db4/1461073718156/RISE-Manifesto-2016.pdf> Accessed 16.1.2023.

BBC News (2016) Holyrood 2016: UKIP manifesto at-a-glance. <https://www.bbc.co.uk/news/election-2015-scotland-35985165> Accessed 16.1.2023.

Scottish Trade Unionist and Socialist Coalition (2021) Core manifesto for the 2021 Scottish Parliament election. <https://www.tusc.org.uk/17486/01-03-2021/core-manifesto-for-the-2021-scottish-parliament-election> Accessed 16.1.2023.

Reform UK Scotland (2021) Building a COVID recovery for Scotland. <https://reformuk.scot/wp-content/uploads/2021/04/Reform-Scotland-Economic-Plan.pdf> Accessed 16.1.2023.

UK Government (2022) Income Tax factsheet 23 September 2022. <https://www.gov.uk/government/publications/the-growth-plan-2022-factsheet-on-income-tax/income-tax-factsheet>. Accessed 16.1.2023.

Appendix 3. Rates of social security payments devolved to the Scottish Government (2022/23).

| **Devolved benefit** | **Means-tested?** | **Rate 2022/23^iii^** | **No. of cases in Scotland^iv^** |
| --- | --- | --- | --- |
| Attendance Allowance | No | Lower £61·85/week, Higher £92·40/week. | 124,081 cases (Feb 2022) |
| Carer’s Allowance^i,ii^ | No | £69·70/week. | 81,059 cases (Feb 2022) |
| Carer’s Allowance Supplement ^i, ii^ | No | £245·70 in June and £245·70 in December (£491·40/year total). | 81,680 cases (Apr 2022) |
| Disability Living Allowance | No | Care Component: Lowest £24·45/week, Middle £61·85/week, Highest £92·40/week. Mobility Component: Lower £24·45/week, Higher £64·50/week. | 138,218 cases (Feb 2022) |
| Industrial Injuries Disablement Benefit ^ii^ | No | Between £37.72/week and £188.60/week, depending on disability | 25,306 cases (2021 Q3) |
| Personal Independence Payment | No | Daily Living Component: Standard £61·85/week, Enhanced £92·40/week. Mobility Component: Standard £24·45/week, Enhanced £64·50/week. | 322,387 cases (Jul 2022) |
| Severe Disablement Allowance ^ii^ | No | £83·75/week, with age-related additions: Lower/middle £6·95/week, Higher £12·55/week. | 1,290 cases (Feb 2022) |
| Best Start Grant | Yes | Three one-off payments per child: Pregnancy and Baby Payment (PBP): £642·35 for first child, £321·20 for subsequent children. Early Learning Payment (ELP): £267·65 per child. School Age Payment (SAP): £267·65 per child. | 2021-22 cases:  PBP 14,935  ELP 14,520  SAP 16,675 |
| Scottish Child Payment | Yes | £20/week per child (0-5 years up to Nov 2022, 0-15 years thereafter). | 104,000 (Jun 2022) |
| Scottish Child Payment Bridging Payment | Yes | Per child over 5 years who qualifies for free school meals due to low household income: April, Summer and October 2022: £130; December 2022: £260. Total: £650/year | Not available (locally administered) |
| Child Winter Heating Assistance | Yes | £214·10 per child or young person (one-off payment). | 19,865 cases (2021/22) |
| Winter Heating Payment | Yes | £50/year. | Not available (starts Feb 2023) |

^i^ Included in taxable income. ^ii^ Included in the income test for means-tested benefits.

^iii^ From [Social Security Scotland](https://www.socialsecurity.gov.scot/about/statistics/social-security-scotland-statistics-publications) and [Department for Work and Pensions](https://stat-xplore.dwp.gov.uk/).

^iv^ From [Scottish Government](https://www.mygov.scot/browse/benefits) and [UK Government](https://www.gov.uk/government/publications/benefit-and-pension-rates-2022-to-2023/proposed-benefit-and-pension-rates-2022-to-2023)

Appendix 4. Household income modelling

We estimated the impact of each policy scenario on household incomes in Scotland in 2022/23. We used the tax-benefit microsimulation model UKMOD (Richiardi et al. 2021) to estimate incomes (before housing costs) for Scottish households in the Family Resources Survey (FRS; a cross-sectional survey of private households; sample size 2704; response rate 56%; Office for National Statistics 2019). FRS data from 2015/16 were used as the area deprivation level (Scottish Index of Multiple Deprivation (SIMD) 2016) for these households was known (via data sharing agreement with Department for Work and Pensions (DWP)), and SIMD was required for linking household data to routine health data.

We ran the models for the financial year 2022/23, with monetary values uprated to 2022/23 where necessary, and benefit uptake rates from the DWP and Her Majesty’s Revenue and Customs (HMRC) (De Agostini 2017). The modelling accounted for how increases to some devolved benefits could affect eligibility for means-tested benefits and increase taxable income. We equivalised household income using the Organisation for Economic Co-operation and Development (OECD) modified equivalence scale (Department for Work and Pensions 2020). In this scale the reference is a couple with no children. National-level results for Scotland were produced using weights that accounted for differential non-response to the FRS (UK Data Archive 2017). We calculated average equivalised household income under each policy, by SIMD quintile.

References:

De Agostini P. EUROMOD Country Report United Kingdom 2013-2016. Colchester, UK: Institute for Social and Economic Research, 2017.

Department for Work and Pensions. Households Below Average Income (HBAI) Quality and Methodology Information Report 2018/19. 2020. https://assets.publishing.service.gov.uk/government/uploads/system/uploads/attachment_data/file/875331/households-below-average-income-quality-methodology-2018-2019.pdf (accessed January 16, 2023).

Office for National Statistics, Social and Vital Statistics Division, Department for Work and Pensions. Family Resources Survey. National Centre for Social Research 2019.

Richiardi M, Collado C, Popova D. UKMOD – A new tax-benefit model for the four nations of the UK. *Int J Microsim* 2021; **14**: 92–101.

UK Data Archive. Introduction to the Family Resources Survey 2015-2016. 2017. http://doc.ukdataservice.ac.uk/doc/8171/mrdoc/pdf/frs_2015_16_introduction_family_resources_survey.pdf (accessed January 16, 2023).

Appendix 5. Effect of household income change on mortality rates

In the absence of empirical evidence of the relationship between income change and mortality we used the relationship between income difference and mortality, from cross-sectional data (Table A1). The dependent variable was all-cause mortality rates (European age-standardised rates (EASRs), calculated using 2015 data from National Records of Scotland (NRS) and the European Standard Population 2013) and the independent variable was mean equivalised household income (before housing costs) for 2015/16 FRS respondents, by SIMD 2016 quintile (Table A1).

The choice of function was driven by the asymptotic shape of the plotted data, and the assumption that the same absolute or relative change in income will have a larger effect on health for lower income than higher income households. A non-linear least squares logistic regression model gave a good fit to the data (Figure A1), with the formula:

$$EASR= \frac{1+ e^{\frac{1826.84-income}{539.41}}}{0.00124}$$

We used this formula to estimate mortality rates associated with the SIMD-level household incomes we had estimated for each policy. Rate ratios for the mortality effect for each policy, by SIMD quintile, were calculated by dividing the policy EASR estimate by the baseline EASR estimate.

We also used this relationship to assess how pre-specified changes in household income would affect mortality rates and life expectancy, and how this would vary by SIMD quintile (with average household incomes as per Table A1). Estimated impacts on life expectancy are shown in Table A2, for household income changes ranging from -6% to +6%. This shows that the same percentage change in income would have a much larger effect on life expectancy in more deprived than in less deprived areas.

**Table A1. SIMD quintile-level household income and mortality rate data used to estimate the relationship between income and mortality.**

| SIMD 2016 quintile | Mean equivalised household income before housing costs (£/month), 2015/16 | Mortality EASR (per 100,000), 2015 |
| --- | --- | --- |
| Q1 (most deprived) | 1,833 | 1,597 |
| Q2 | 2,085 | 1,324 |
| Q3 | 2,269 | 1,172 |
| Q4 | 2,497 | 1,033 |
| Q5 (least deprived) | 3,084 | 890 |

Source: Weighted averages of household income data for 2015/16 Scottish FRS respondents linked to SIMD 2016 quintiles (DWP mapping); NRS population and deaths data. EASRs standardised to the 2013 European Standard Population.

**Figure A1. Logistic relationship between household income and mortality rate.**

Red dots show the SIMD-level data points used to fit the model (Table A1), and the black line shows the fitted model predictions.


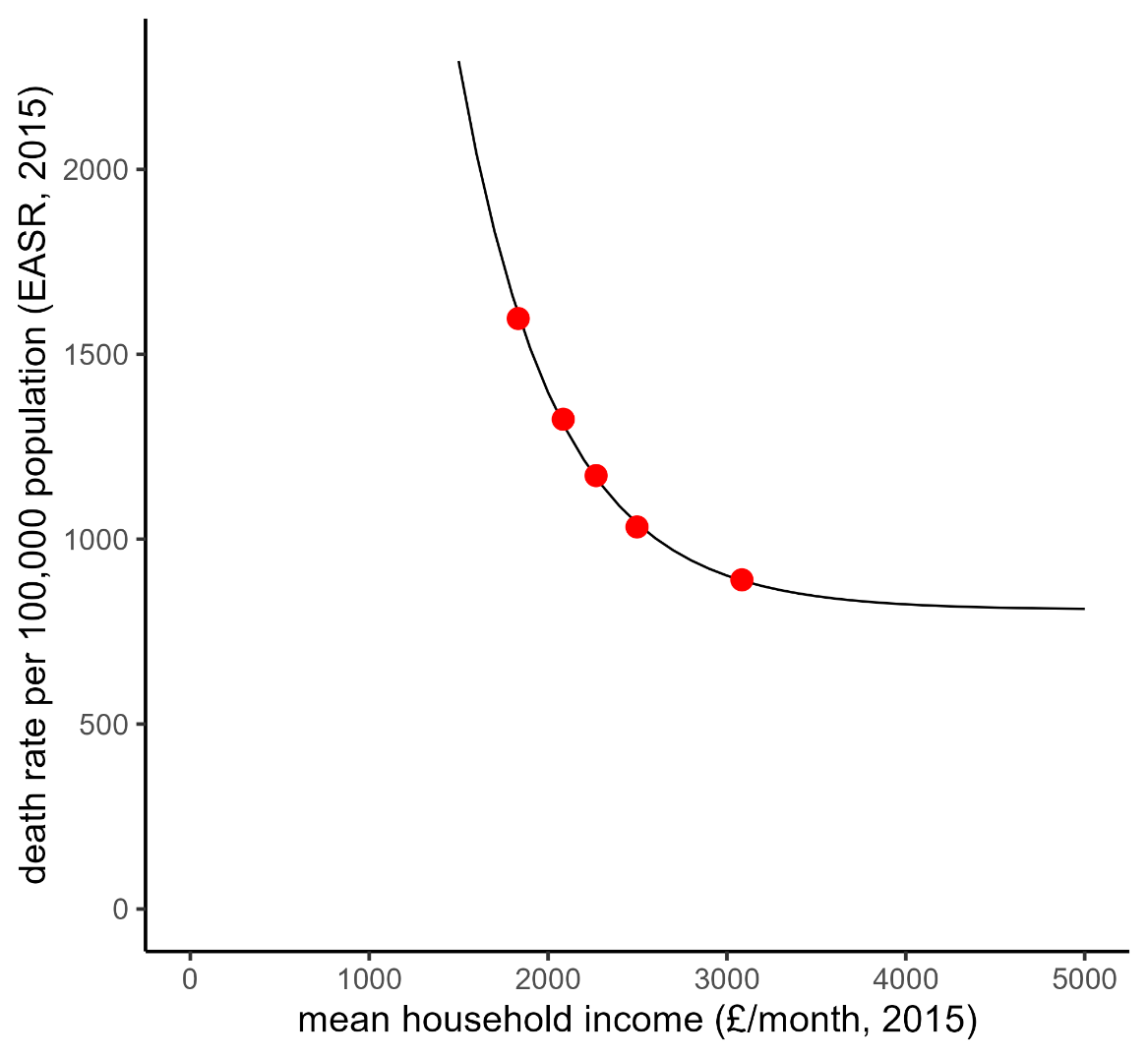


**Table A2. Change in life expectancy (%) predicted for a range of household income changes (%), by SIMD quintile.**

|  | Change in household income (%) | | | | | | |
| --- | --- | --- | --- | --- | --- | --- | --- |
| SIMD 2016 quintile | -6% | -4% | -2% | 0% | 2% | 4% | 6% |
| 1 (most deprived) | -1.28 | -0.83 | -0.40 | 0.00 | 0.38 | 0.74 | 1.08 |
| 2 | -0.87 | -0.56 | -0.27 | 0.00 | 0.25 | 0.48 | 0.70 |
| 3 | -0.65 | -0.41 | -0.20 | 0.00 | 0.18 | 0.35 | 0.50 |
| 4 | -0.44 | -0.28 | -0.13 | 0.00 | 0.12 | 0.23 | 0.32 |
| 5 (least deprived) | -0.17 | -0.11 | -0.05 | 0.00 | 0.04 | 0.08 | 0.12 |
| Overall | -0.73 | -0.47 | -0.22 | 0.00 | 0.21 | 0.40 | 0.57 |

Appendix 6. Estimated direct net fiscal costs of the scenarios, relative to baseline.

For each scenario we used UKMOD to calculate the direct fiscal costs of payments to households (social security) and fiscal revenues from households (taxes and National Insurance Contributions (NIC)). We used FRS survey weights to gross these up to the Scottish population, and calculated the net cost of each scenario relative to baseline.

| Scenario | Net fiscal cost  (£ billion p.a.) |
| --- | --- |
| Baseline | 0 |
| Low 1 | 5,567 |
| Low 2 | 2,174 |
| Low 3 | 844 |
| Low 4 | 818 |
| Mid 1 | 414 |
| Mid 2 | 211 |
| Mid 3 | -38 |
| Mid 4 | -164 |
| High 1 | -281 |
| High 2 | -576 |
| High 3 | -837 |
| High 4 | -2,661 |
| Devolved benefits + 10% | 239 |
| Devolved benefits + 25% | 725 |
| Devolved benefits + 50% | 1,528 |
| Devolved benefits + 75% | 2,336 |
| Devolved benefits + 100% | 3,143 |
| High 4 + Devolved benefits + 10% | -2,942 |
| High 4 + Devolved benefits + 25% | -2,456 |
| High 4 + Devolved benefits + 50% | -1,653 |
| High 4 + Devolved benefits + 75% | -844 |
| High 4 + Devolved benefits + 100% | -37 |

Appendix 7. Life expectancy at birth, and inequalities in life expectancy at birth, in the baseline scenario (2022/23).

|  | **Female** | **Male** |
| --- | --- | --- |
| **Life expectancy at birth (years)** |  |  |
| Scottish population | 80·6 | 76·5 |
| Most deprived 20% (SIMD quintile 1) | 75·5 | 69·8 |
| Least deprived 20% (SIMD quintile 5) | 84·8 | 81·6 |
| **Inequalities in life expectancy at birth** |  |  |
| Absolute inequality (SII, years)^i^ | 11·68 | 15·14 |
| Relative inequality (RII)^ii^ | 0·14 | 0·20 |

^i^ SII, Slope Index of Inequality.

^ii^ RII, Relative Index of Inequality

Appendix 8. Differences in life expectancy and inequalities in life expectancy from the baseline scenario, by sex.

Negative changes indicate a narrowing of inequalities.

|  | Female | | | | | Male | | | | |
| --- | --- | --- | --- | --- | --- | --- | --- | --- | --- | --- |
|  | Inequality difference (%) | | Life expectancy difference (weeks) | | | Inequality difference (%) | | Life expectancy difference (weeks) | | |
| Scenario | Absolute (SII) | Relative (RII) | SIMD Q1 | SIMD Q5 | Overall | Absolute (SII) | Relative (RII) | SIMD Q1 | SIMD Q5 | Overall |
| Low 1 | -4·6 | -5·1 | 30·1 | 6·6 | 21·0 | -4·1 | -4·7 | 33·6 | 6·8 | 23·3 |
| Low 2 | -0·9 | -1·1 | 8·4 | 3·5 | 7·5 | -0·9 | -1·1 | 9·4 | 3·6 | 8·2 |
| Low 3 | -0·2 | -0·3 | 2·9 | 1·5 | 2·8 | -0·2 | -0·3 | 3·3 | 1·5 | 3·1 |
| Low 4 | -0·2 | -0·3 | 2·8 | 1·5 | 2·7 | -0·2 | -0·3 | 3·1 | 1·5 | 3·0 |
| Mid 1 | -0·1 | -0·2 | 1·5 | 0·8 | 1·4 | -0·1 | -0·2 | 1·7 | 0·8 | 1·5 |
| Mid 2 | 0·0 | 0·0 | 0·7 | 0·4 | 0·7 | 0·0 | -0·1 | 0·7 | 0·4 | 0·7 |
| Mid 3 | 0·0 | 0·0 | -0·2 | -0·1 | -0·2 | 0·0 | 0·0 | -0·2 | -0·1 | -0·2 |
| Mid 4 | 0·3 | 0·3 | -1·7 | -0·1 | -1·0 | 0·3 | 0·3 | -1·9 | -0·1 | -1·1 |
| High 1 | 0·3 | 0·4 | -2·2 | -0·4 | -1·3 | 0·3 | 0·3 | -2·5 | -0·4 | -1·5 |
| High 2 | 0·0 | 0·0 | -1·9 | -1·6 | -1·5 | 0·0 | 0·1 | -2·1 | -1·6 | -1·6 |
| High 3 | 0·1 | 0·2 | -3·0 | -2·0 | -2·5 | 0·1 | 0·2 | -3·3 | -2·1 | -2·7 |
| High 4 | -0·5 | -0·3 | -7·4 | -8·2 | -7·4 | -0·3 | -0·1 | -8·3 | -8·4 | -8·0 |
| Devolved + 10% | -0·8 | -0·8 | 4·0 | 0·1 | 1·6 | -0·7 | -0·7 | 4·4 | 0·1 | 1·9 |
| Devolved + 25% | -2·3 | -2·4 | 11·4 | 0·3 | 4·9 | -2·0 | -2·1 | 12·7 | 0·3 | 5·5 |
| Devolved + 50% | -4·6 | -4·9 | 23·3 | 0·6 | 10·0 | -4·0 | -4·3 | 26·0 | 0·6 | 11·3 |
| Devolved + 75% | -6·9 | -7·2 | 34·7 | 0·9 | 14·8 | -6·0 | -6·4 | 38·7 | 1·0 | 16·8 |
| Devolved + 100% | -9·1 | -9·5 | 45·4 | 1·3 | 19·4 | -7·9 | -8·4 | 50·7 | 1·3 | 22·0 |
| High4 + Devolved+10% | -0·6 | -0·4 | -7·5 | -9·0 | -8·5 | -0·4 | -0·2 | -8·4 | -9·2 | -9·2 |
| High4 + Devolved+25% | -2·2 | -2·1 | 0·3 | -8·7 | -5·0 | -1·7 | -1·6 | 0·3 | -8·9 | -5·3 |
| High4 + Devolved+50% | -4·6 | -4·6 | 12·7 | -8·2 | 0·4 | -3·9 | -3·9 | 14·2 | -8·3 | 0·9 |
| High4 + Devolved+75% | -6·9 | -7·1 | 24·6 | -7·7 | 5·7 | -5·9 | -6·1 | 27·4 | -7·8 | 6·8 |
| High4 + Devolved+100% | -9·2 | -9·4 | 35·8 | -7·2 | 10·6 | -7·8 | -8·1 | 40·0 | -7·3 | 12·4 |

SII, Slope Index of Inequality; RII, Relative Index of Inequality; SIMD, Scottish Index of Multiple Deprivation; Q1, most deprived quintile; Q5, least deprived quintile.

Appendix 9. Implications of the policy scenarios for net cost to government and female life expectancy inequality.

a) Income tax policies

**
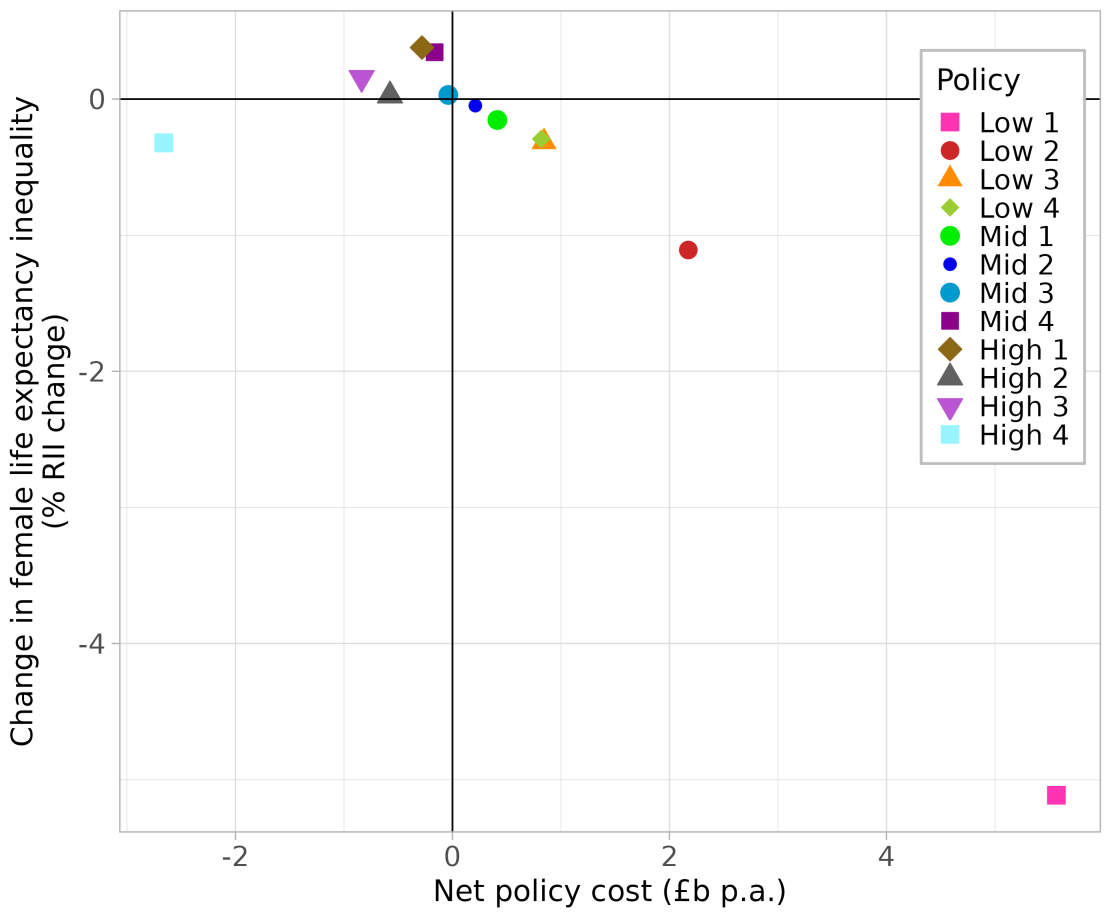
**

b) Devolved benefits increases


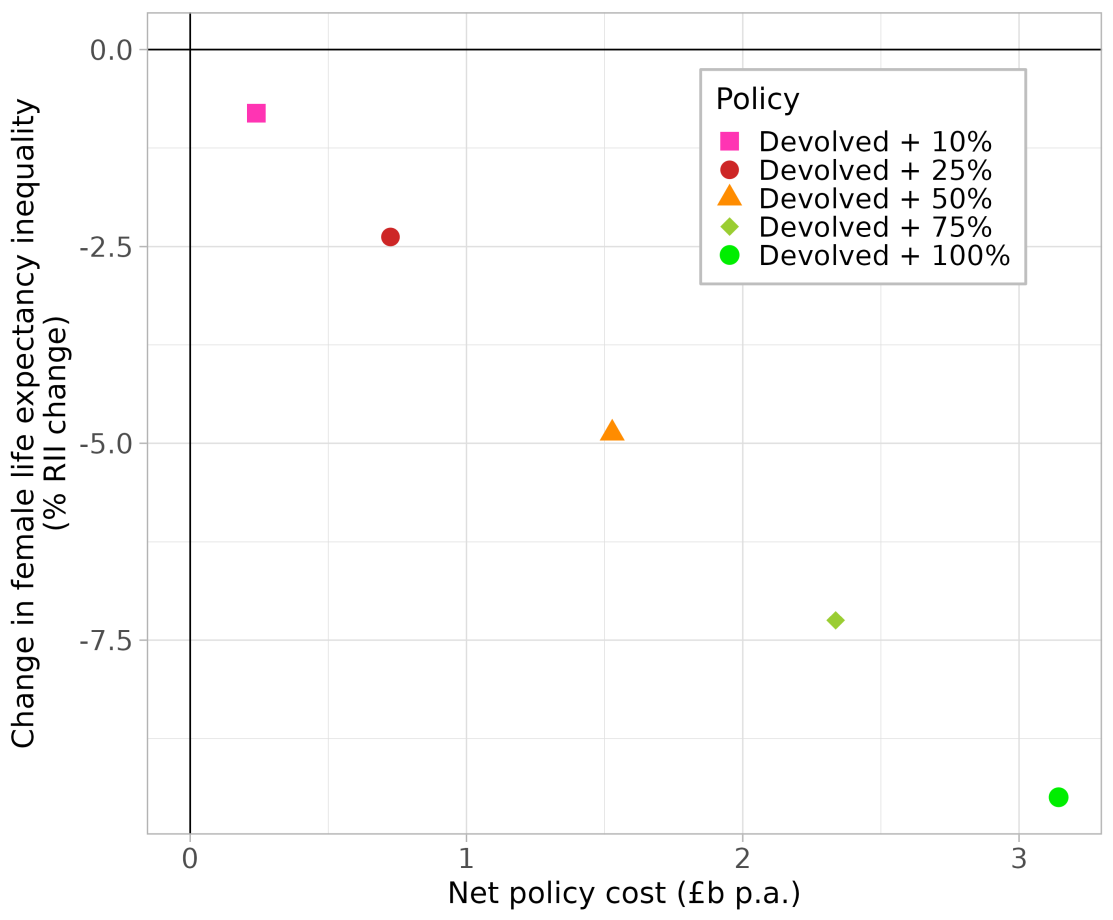


Appendix 10. Difference from baseline in scenario cost and income effects, for “High 4” and devolved benefits increases separately and in combination.

|  | Change in fiscal cost  (£b p.a.) | Change in real household income (%) by SIMD quintile | | | | | Change in income inequality (% Gini) |
| --- | --- | --- | --- | --- | --- | --- | --- |
| Scenario |  | Q1 | Q2 | Q3 | Q4 | Q5 |  |
| High 4 | -2·66 | -0·9 | -0·9 | -1·6 | -3·3 | -6·3 | -6·5 |
| Devolved + 10% | 0·24 | 0·5 | 0·3 | 0·3 | 0·2 | 0·1 | -0·5 |
| Devolved + 25% | 0·72 | 1·5 | 1·1 | 0·8 | 0·5 | 0·3 | -1·3 |
| Devolved + 50% | 1·53 | 3·2 | 2·3 | 1·6 | 1·1 | 0·6 | -2·4 |
| Devolved + 75% | 2·34 | 4·8 | 3·5 | 2·4 | 1·6 | 0·9 | -3·3 |
| Devolved + 100% | 3·14 | 6·5 | 4·8 | 3·2 | 2·2 | 1·3 | -3·9 |
| High 4 + Devolved+10% | -2·94 | -1·0 | -1·2 | -2·0 | -3·8 | -6·8 | -7·1 |
| High 4 + Devolved+25% | -2·46 | 0·0 | -0·5 | -1·5 | -3·4 | -6·6 | -7·9 |
| High 4 + Devolved+50% | -1·65 | 1·7 | 0·7 | -0·7 | -2·9 | -6·3 | -8·9 |
| High 4 + Devolved+75% | -0·84 | 3·3 | 2·0 | 0·1 | -2·3 | -6·0 | -9·7 |
| High 4 + Devolved+100% | -0·04 | 5·0 | 3·2 | 1·0 | -1·7 | -5·7 | -10·3 |

SIMD, Scottish Index of Multiple Deprivation; Q1, most deprived quintile; Q5, least deprived quintile.

Appendix 11. Difference from baseline in scenario life expectancy and life expectancy inequality effects, for “High 4” and devolved benefits increases separately and in combination.

|  | Change in life expectancy (weeks) by SIMD quintile | | | | | | Change in relative inequality (RII, %) | |
| --- | --- | --- | --- | --- | --- | --- | --- | --- |
|  | Female | | | Male | | | Female | Male |
| Scenario | Q1 | Q5 | Overall | Q1 | Q5 | Overall |  |  |
| High 4 | -7·4 | -8·2 | -7·4 | -8·3 | -8·4 | -8·0 | -0·3 | -0·1 |
| Devolved + 10% | 4·0 | 0·1 | 1·6 | 4·4 | 0·1 | 1·9 | -0·8 | -0·7 |
| Devolved + 25% | 11·4 | 0·3 | 4·9 | 12·7 | 0·3 | 5·5 | -2·4 | -2·1 |
| Devolved + 50% | 23·3 | 0·6 | 10·0 | 26·0 | 0·6 | 11·3 | -4·9 | -4·3 |
| Devolved + 75% | 34·7 | 0·9 | 14·8 | 38·7 | 1·0 | 16·8 | -7·2 | -6·4 |
| Devolved + 100% | 45·4 | 1·3 | 19·4 | 50·7 | 1·3 | 22·0 | -9·5 | -8·4 |
| High 4 + Devolved+10% | -7·5 | -9·0 | -8·5 | -8·4 | -9·2 | -9·2 | -0·4 | -0·2 |
| High 4 + Devolved+25% | 0·3 | -8·7 | -5·0 | 0·3 | -8·9 | -5·3 | -2·1 | -1·6 |
| High 4 + Devolved+50% | 12·7 | -8·2 | 0·4 | 14·2 | -8·3 | 0·9 | -4·6 | -3·9 |
| High 4 + Devolved+75% | 24·6 | -7·7 | 5·7 | 27·4 | -7·8 | 6·8 | -7·1 | -6·1 |
| High 4 + Devolved+100% | 35·8 | -7·2 | 10·6 | 40·0 | -7·3 | 12·4 | -9·4 | -8·1 |

RII, Relative Index of Inequality; SIMD, Scottish Index of Multiple Deprivation; Q1, most deprived quintile; Q5, least deprived quintile.

Appendix 12. Differences in life expectancy and inequalities in life expectancy from the baseline scenario, by sex, if the income-mortality relationship was weakened by 50%.

Negative changes indicate a narrowing of inequalities.

|  | Female | | | | | Male | | | | |
| --- | --- | --- | --- | --- | --- | --- | --- | --- | --- | --- |
|  | Inequality difference (%) | | Life expectancy difference (weeks) | | | Inequality difference (%) | | Life expectancy difference (weeks) | | |
| Scenario | Absolute (SII) | Relative (RII) | SIMD Q1 | SIMD Q5 | Overall | Absolute (SII) | Relative (RII) | SIMD Q1 | SIMD Q5 | Overall |
| Low 1 | -2·3 | -2·5 | 14·9 | 3·3 | 10·4 | -2·0 | -2·3 | 16·6 | 3·4 | 11·5 |
| Low 2 | -0·5 | -0·6 | 4·2 | 1·8 | 3·7 | -0·4 | -0·5 | 4·7 | 1·8 | 4·1 |
| Low 3 | -0·1 | -0·2 | 1·5 | 0·8 | 1·4 | -0·1 | -0·2 | 1·6 | 0·8 | 1·6 |
| Low 4 | -0·1 | -0·1 | 1·4 | 0·7 | 1·4 | -0·1 | -0·1 | 1·6 | 0·8 | 1·5 |
| Mid 1 | -0·1 | -0·1 | 0·7 | 0·4 | 0·7 | -0·1 | -0·1 | 0·8 | 0·4 | 0·8 |
| Mid 2 | 0·0 | 0·0 | 0·3 | 0·2 | 0·3 | 0·0 | 0·0 | 0·4 | 0·2 | 0·4 |
| Mid 3 | 0·0 | 0·0 | -0·1 | 0·0 | -0·1 | 0·0 | 0·0 | -0·1 | 0·0 | -0·1 |
| Mid 4 | 0·2 | 0·2 | -0·9 | -0·1 | -0·5 | 0·1 | 0·2 | -1·0 | -0·1 | -0·6 |
| High 1 | 0·2 | 0·2 | -1·1 | -0·2 | -0·7 | 0·2 | 0·2 | -1·3 | -0·2 | -0·7 |
| High 2 | 0·0 | 0·0 | -0·9 | -0·8 | -0·7 | 0·0 | 0·0 | -1·1 | -0·8 | -0·8 |
| High 3 | 0·0 | 0·1 | -1·5 | -1·0 | -1·3 | 0·1 | 0·1 | -1·7 | -1·0 | -1·4 |
| High 4 | -0·3 | -0·2 | -3·7 | -4·1 | -3·7 | -0·1 | 0·0 | -4·2 | -4·2 | -4·0 |
| Devolved + 10% | -0·4 | -0·4 | 2·0 | 0·0 | 0·8 | -0·3 | -0·4 | 2·2 | 0·0 | 0·9 |
| Devolved + 25% | -1·1 | -1·2 | 5·7 | 0·1 | 2·4 | -1·0 | -1·0 | 6·3 | 0·1 | 2·7 |
| Devolved + 50% | -2·3 | -2·4 | 11·5 | 0·3 | 5·0 | -2·0 | -2·1 | 12·9 | 0·3 | 5·6 |
| Devolved + 75% | -3·4 | -3·6 | 17·1 | 0·5 | 7·4 | -3·0 | -3·2 | 19·1 | 0·5 | 8·4 |
| Devolved + 100% | -4·4 | -4·7 | 22·3 | 0·6 | 9·6 | -3·9 | -4·1 | 24·9 | 0·6 | 10·9 |
| High4 + Devolved+10% | -0·3 | -0·2 | -3·8 | -4·5 | -4·2 | -0·2 | -0·1 | -4·2 | -4·6 | -4·6 |
| High4 + Devolved+25% | -1·1 | -1·0 | 0·1 | -4·4 | -2·5 | -0·9 | -0·8 | 0·1 | -4·5 | -2·6 |
| High4 + Devolved+50% | -2·3 | -2·3 | 6·3 | -4·1 | 0·2 | -1·9 | -1·9 | 7·1 | -4·2 | 0·5 |
| High4 + Devolved+75% | -3·5 | -3·5 | 12·1 | -3·8 | 2·8 | -2·9 | -3·0 | 13·6 | -3·9 | 3·4 |
| High4 + Devolved+100% | -4·5 | -4·6 | 17·6 | -3·6 | 5·3 | -3·9 | -4·0 | 19·7 | -3·7 | 6·2 |

SII, Slope Index of Inequality; RII, Relative Index of Inequality; SIMD, Scottish Index of Multiple Deprivation; Q1, most deprived quintile; Q5, least deprived quintile.
